# Post-treatment Lyme Disease as a Model for Persistent Symptoms in Lyme Disease

**DOI:** 10.3389/fmed.2020.00057

**Published:** 2020-02-25

**Authors:** Alison W. Rebman, John N. Aucott

**Affiliations:** Lyme Disease Research Center, Division of Rheumatology, Department of Medicine, Johns Hopkins University School of Medicine, Baltimore, MD, United States

**Keywords:** Lyme disease, post-treatment Lyme disease, review, clinical presentation, research priorities

## Abstract

It has long been observed in clinical practice that a subset of patients with Lyme disease report a constellation of symptoms such as fatigue, cognitive difficulties, and musculoskeletal pain, which may last for a significant period of time. These symptoms, which can range from mild to severe, have been reported throughout the literature in both prospective and population-based studies in Lyme disease endemic regions. The etiology of these symptoms is unknown, however several illness-causing mechanisms have been hypothesized, including microbial persistence, host immune dysregulation through inflammatory or secondary autoimmune pathways, or altered neural networks, as in central sensitization. Evaluation and characterization of persistent symptoms in Lyme disease is complicated by potential independent, repeat exposures to *B. burgdorferi*, as well as the potential for co-morbid diseases with overlapping symptom profiles. Antibody testing for *B. burgdorferi* is an insensitive measure after treatment, and no other FDA-approved tests currently exist. As such, diagnosis presents a complex challenge for physicians, while the lived experience for patients is one marked by uncertainty and often illness invalidation. Currently, there are no FDA-approved pharmaceutical therapies, and the safety and efficacy of off-label and/or complementary therapies have not been well studied and are not agreed-upon within the medical community. Post-treatment Lyme disease represents a narrow, defined, mechanistically-neutral subset of this larger, more heterogeneous group of patients, and is a useful definition in research settings as an initial subgroup of study. The aim of this paper is to review the current literature on the diagnosis, etiology, risk factors, and treatment of patients with persistent symptoms in the context of Lyme disease. The meaning and relevance of existing patient subgroups will be discussed, as will future research priorities, including the need to develop illness biomarkers, elucidate the biologic mechanisms of disease, and drive improvements in therapeutic options.

## Background

Lyme disease is a geographically expanding, vector-borne disease which is transmitted to humans through the bite of a tick infected with various genospecies of the spirochete bacteria *B. burgdorferi* sensu lato (1, 2). The species of *Ixodes* ticks which transmit the disease are commonly found throughout temperate regions of North America, Europe, and Asia (2). Currently, the Centers for Disease Control and Prevention (CDC) estimate approximately 300,000 new cases of Lyme disease in the United States alone each year (3). However, due to climate change, shifting land use patterns, and the relative abundance and distribution of reservoir hosts, it is anticipated that the geographic range of the tick vector will continue to expand (4, 5). For instance, the number of reported cases in Canada has increased six-fold over the past decade, with particular increases in the eastern provinces of Nova Scotia and Ontario (6, 7).

Clinically, Lyme disease presents with dermatologic and/or viral-like signs and symptoms such as intermittent fever, sweats, chills, malaise, fatigue, and achiness during the acute phase, which can transition to neurologic, cardiac, and/or joint involvement in later stages of the infection as the bacteria disseminate hematogenously (8). Along with these objective signs, persistent and recurrent symptoms such as fatigue, sleep disruption, arthralgia, myalgia, and headache are also commonly present during later stages of untreated Lyme disease and may account for the majority of the patient symptom experience (9). For example, patients with intermittent bouts of late Lyme arthritis continued to have such symptoms present during the intervening intervals (10). Occasionally, symptoms without physical exam, laboratory, or other so-called “objective” findings remain the major or only manifestations of untreated Lyme disease infection (11). The use of direct tests such as culture, polymerase chain reaction (PCR) or antigen detection for *B. burgdorferi* to aid clinicians in diagnosis is extremely limited, and *B. burgdorferi* cannot be cultured in non-research settings. A two-tier antibody test is widely available and utilized despite significant sensitivity limitations, particularly in early infection and in the convalescent phase after antibiotic treatment of early Lyme disease (12, 13). All stages of Lyme disease are currently treated with antibiotics (14).

The majority of patients return to their pre-morbid health following recommended antibiotic treatment for Lyme disease. However, it has long been observed in clinical practice and in research settings that a subset of patients continue to report a constellation of largely patient reported, so-called “subjective” symptoms which may last for a significant period of time following treatment (15–24). Nevertheless, the epidemiology, significance, etiology, and appropriate treatment of these persistent symptoms are not well-understood and as such, remain the subject of a great deal of scientific dispute and controversy within the medical community (25–28). Patients who can be said to have post-treatment Lyme disease (PTLD) (also called post-treatment Lyme disease syndrome or post-Lyme disease syndrome) represent a narrow, highly specific subset of the broader population of patients with persistent symptoms (14). This specificity is important in research, but not always in clinical settings, as there are multiple pathways through which patients who may be suffering from on-going symptoms from Lyme disease may not meet these narrow criteria. The term PTLD is neutral to underlying disease mechanism and as such, we do not necessarily assume that patients with PTLD have achieved microbiologic cure with initial antibiotic therapy. The aim of this manuscript is to review the published literature from a variety of academic disciplines and perspectives on symptoms which persist or recur in the setting of Lyme disease. We acknowledge that this is a broad topic, and one limitation of our manuscript is that not all related concepts could be readily addressed due to space constraints.

## Estimated Frequency

After Lyme disease was first identified in the United States in the late 1970's, but before the pathogenic bacteria was recognized, it was noted that untreated patients with Lyme arthritis often also reported concurrent symptoms such as headache, fatigue, myalgia, and hyperesthesia (9). It was first reported in some of the earliest cases series of treated patients that these symptoms could persist following antibiotic therapy (29, 30). Among patients diagnosed and treated in the early to mid-1980's, largely with penicillin and/or tetracycline, up to 50% experienced symptoms such as fatigue, musculoskeletal pain, memory impairment, and headache several years after treatment (29, 31, 32). A large, population-based study on Nantucket Island found that 36% of those with Lyme disease contracted and treated in the late 1980's had on-going symptoms six years later, and that they were significantly more likely than those without a history of Lyme disease to report fatigue, headache, cognitive complaints, sleep disturbance, and musculoskeletal pain, numbness and/or weakness (33).

As more effective antibiotic treatments and drug regimens were tested and identified in prospective studies and clinical treatment trials, other investigators reported estimates of 0 to 35% for persistent, non-specific symptoms following treatment (15–24). These symptoms were often considered “minor” and classified independently from defined treatment failure; objective signs of neurologic, cardiac, or joint involvement which would indicate progression to later stages of the infection. This relatively broad range of estimates is likely a reflection of several of the study design challenges which are still relevant in the field today. First, inter-study variability in enrollment criteria may encompass factors directly related to risk of persistent symptoms (see section Risk Factors). For instance, studies which require an active erythema migrans (EM) rash at enrollment will by definition exclude patients with longer disease durations, a likely risk factor for persistent symptoms. Population-based studies may be more reflective of the community practice of medicine than those conducted in academic research centers, with a wider range of treatment regimens, a higher misdiagnosis rate, and longer duration of disease prior to appropriate antibiotic treatment. Finally, without an objective biomarker, there has been a lack of standardization in outcome ascertainment, with many studies relying on physician assessment and classification into subjective sub-categories.

While Lyme disease has been a nationally notifiable disease in the United States since 1991, the CDC does not track disease outcomes or cases of persistent symptoms (34). Estimating the population-level prevalence of persistent symptoms following Lyme disease is challenging due to this lack of standardization or consensus in operationalizing a case definition. Furthermore, it is hindered by the difficulty of obtaining valid incidence rate estimates of new Lyme disease infections, as Lyme disease has traditionally been tracked through passive surveillance which has historically led to significant under-reporting of cases (35). One recent study attempted to estimate cumulative prevalence of persistent symptoms after treatment using statistical simulation techniques (36). The authors estimate almost 1 million cases by 2020, assuming continued linear growth of new Lyme disease cases since 1980 and a potentially conservative 10% “failure” rate of new infections.

## Risk Factors

Several clinical factors surrounding the initial onset of Lyme disease have been found to increase risk for persistent symptoms after treatment. More severe disease at onset in the form of a higher number of symptoms (37) and/or objective signs (such as Bell's Palsy) or symptoms (such as headache, photophobia, or neck pain) which suggest dissemination to the nervous system may be present and may increase the risk of persistent symptoms following treatment (33, 38). One recent study has shown that the presence of pre-existing co-morbidities in Lyme disease was predictive of long-term symptoms and lower quality of life (39), similar to other disease settings (40, 41). Delays in diagnosis and initiation of appropriate treatment, which importantly may be driven by patient health insurance status (42), have also been shown to increase risk (31, 32). Diagnostic delays may also be compounded in some initially misdiagnosed patients by subsequent exposure to inappropriate or ineffective treatments (15, 43). While it is unknown whether corticosteroid exposure during acute infection, often prescribed for associated facial palsies, may affect resolution of systemic symptoms, it has been shown to be associated with worse long-term facial function outcomes (44, 45). Although awareness of Lyme disease has increased in recent decades, the wide range of clinical heterogeneity at presentation and the limited sensitivity of the two-tier test mean that misdiagnosis and delays in diagnosis still occur with some frequency in the community practice of medicine (42, 43).

Several studies have also suggested that factors relating to the initial immune response to infection may drive later clinical outcomes after treatment. A muted immune response during acute infection, in the form of lower levels of circulating plasmablasts, has been associated with persistent symptoms after treatment (46). However, elevated levels of specific immune mediators such as IL-23 and CCL19 at disease onset and/or in the immediate convalescent period have been associated with the presence of persistent symptoms up to 1 year following treatment (47, 48). It is unclear whether the magnitude of the initial antibody response to *B. burgdorferi* prior to treatment is of importance, as a negative serology has been found to be both associated and not associated with subsequent clinical outcomes (15, 37).

Additionally, while the detailed biology of *B. burgdorferi* is a complex topic (49) which is outside the scope of this article, it has also been hypothesized that specific microbiologic factors may influence treatment outcomes, as over 50 distinct genotypes of *B. burgdorferi* senso stricto have been identified across North America and Europe (50). While associations between infecting genotype and early disseminated disease have been identified (51, 52), more research is needed on potential associations with treatment outcomes. One small study of 14 patients did not find a pattern in the specific genotypes of the infecting *B. burgdorferi* strain among patients with persistent symptoms a decade after treatment for Lyme disease (24). However, RST1 strains which have been found to be more highly inflammatory are associated with more severe symptoms and increased risk of antibiotic-refractory arthritis (53, 54). Furthermore, *Ixodes* ticks are capable of simultaneously carrying and transmitting multiple genotypes of *B. burgdorferi* (55, 56), as well as multiple distinct pathogens. The effects of both of these on risk of persistent symptoms after treatment have not been studied comprehensively, however they may worsen treatment outcomes. In one study, patients co-infected with *B. burgdorferi* and *Babesia microti* experienced not only a greater number and diversity of symptoms, but also took longer to resolve both the signs and symptoms of their illness as well as to be clear of spirochete-specific DNA on PCR testing (57).

Among CDC-reported cases of new Lyme disease infections, there is a slight majority male and a bimodal age distribution among younger children and older adults (58). Similarly, there does not appear to be a significant difference by gender among those who meet a highly-specific definition for persistent symptoms in the research setting (59, 60). However, it has been noted that when less specific definitions are used, as may be applied in clinical practice, the ratio of patients is instead majority female (11, 61). When diagnosed and treated promptly, children appear less likely than adults to report persistent symptoms following Lyme disease (22, 62–64) Among adults, it is not clear if age represents a significant risk factor. Among a sample of culture-confirmed patients with EM, those 50 and older at onset of Lyme disease were not significantly more likely than those under 50 to later report persistent symptoms (65). However, in a recent insurance claims analysis of a large, integrated health system in Pennsylvania, members who met a definition for persistent symptoms following an incident Lyme disease diagnosis were more likely to be older (and female) than those who did not (66).

## Clinical Presentations

Currently, there are no commonly agreed-upon symptoms, laboratory, or imaging findings which are sensitive and specific to aid in the clinical evaluation of patients with persistent symptoms in Lyme disease. Therefore, the clinical diagnosis is primarily one of exclusion, and the current illness must be distinguished both from other systemic inflammatory, rheumatic, malignant and infectious conditions, as well as the effects of co-morbid or pre-existing conditions (39). This raises the possibility of anchoring bias, or the misattribution of either symptoms or positive serologies in low-endemic areas to prior Lyme disease, when in fact the symptoms are caused by a new, unrelated illness (67, 68). While anchoring bias may theoretically play a role in evaluation of patients with persistent symptoms, the extent of its potential contribution is unknown. Furthermore, care should be taken to differentiate prolonged, persistent symptoms from a new, distinct exposure to *B. burgdorferi*, which is often accompanied by a new EM (69). Finally, as the presence of symptoms alone cannot currently definitively establish the link to prior Lyme disease, a second key component is grounding the current illness to the initial exposure to *B. burgdorferi*. This requires a careful, clinical history for clues in the past medical history that may have been missed, such as a misdiagnosed skin lesion or a non-specific, acute, summer, flu-like illness at the onset of the patient's change in health (70, 71). Above all, a thorough clinical history must also account for inter-personal variability, the potential implications of initial misdiagnosis, and the diagnostic limitations of two-tier testing.

### Symptoms

**Figure 1:**
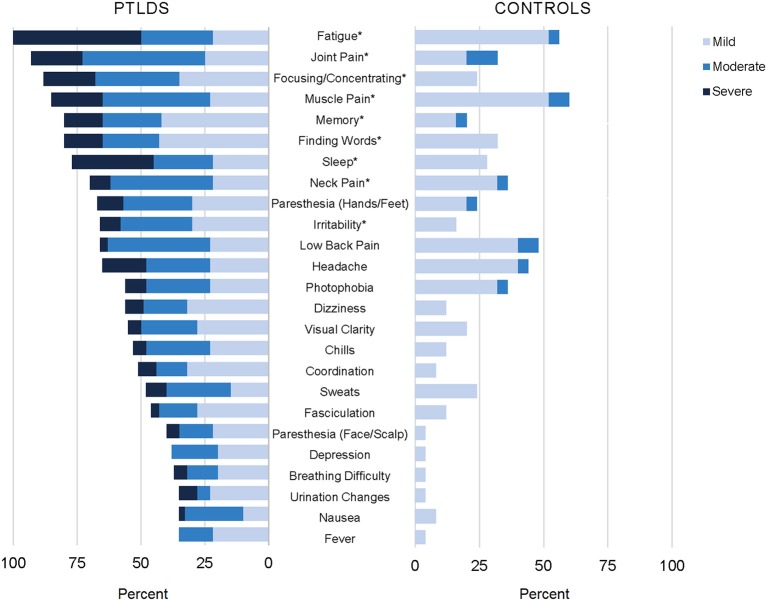
Participants with post-treatment Lyme disease syndrome (PTLDS) and controls were asked about presence and severity of 36 signs/symptoms over the past 2 weeks. Displayed are the 25 signs/symptoms with a statistically significant difference in severity by group (*p* < 0.05), ordered by frequency within the PTLDS group. The nine signs/symptoms with a statistically significant difference at the *p* < 0.001 level are indicated with an asterisk. This figure was originally published and is reprinted from (60) under the Creative Commons CC-BY license.

The prolonged, subjective symptoms frequently reported in the context of Lyme disease (e.g., fatigue, widespread pain, cognitive complaints, paresthesia, and sleep disruption) also broadly represent those commonly reported in outpatient settings (72–74). Furthermore, while some differences in impairment and symptom distribution have been studied and reported (75–77), there is also a degree of general symptom overlap with other disease states such as traumatic brain injury, depression, chronic fatigue syndrome, and fibromyalgia (78). This lack of sensitivity can lead to the conclusion that the prolonged symptoms reported in Lyme disease are no different than the “background noise” of symptoms in the general population. However, the magnitude of the symptoms, as well as the number of co-occurring symptoms reported, is often more severe. In our study of participants with well-characterized PTLD compared to a control group with similar age and gender characteristics, 25 of 36 symptoms assessed were found to be statistically significantly more severe in participants with PTLD (Figure 1) (60). Health-related quality of life, as measured by the 36-item short-form health survey (79), is typically not only lower than controls, but comparable to other major chronic diseases, such as congestive heart failure (60, 80, 81).

Prolonged, persistent symptoms in Lyme disease are primarily patient reported and are therefore considered subjective. Although objective signs may be present, they are not distributed consistently enough across patients nor are they specific enough to be considered diagnostic. Fatigue is often the most commonly reported, severe symptom with levels comparable to patients with multiple sclerosis on the Fatigue Severity Scale instrument (60, 82, 83). In one study, fatigue was also found to be the most important contributor to levels of physical functioning (81). This suggests that fatigue may be an important primary intervention target for patients, including evaluation of related factors such as sleep quality and mood disturbance. Additionally, a history of orthostatic intolerance may be indicative of postural orthostatic tachycardia syndrome or other autonomic dysfunction, which may be another treatable cause of fatigue (84).

Persistent chronic pain in Lyme disease has been described in various studies as neuropathic or nociceptive (85). It has been noted in the literature that pain among patients with PTLD is uniquely asymmetrical, is more likely to involve the limbs, and is less widespread than in fibromyalgia (86, 87), however to our knowledge these observations have not been systematically examined. Pain and/or stiffness in the neck appears to be a common specific presenting location (60, 88). While also meeting criteria for later fibromyalgia may be rare among patients treated promptly for early Lyme disease as a whole (1%) (87), earlier studies suggested that this overlap may be more common among the subset of patients with prolonged, persistent symptoms (89–91). Neurocognitive complaints, including both behavioral and memory-related issues, are also among the most frequently reported symptoms (33, 60, 92).

Although fatigue, musculoskeletal pain, and cognitive dysfunction are the most commonly reported symptoms, a host of others, including sleep disruption, paresthesia, headache, dizziness, and mood changes, are variably distributed across patients (60, 88, 93). As a whole, patients report that symptoms may wax and wane, or may persist with stable levels of severity. While symptoms in some patients may resolve in the initial convalescent period following treatment, for others they may last for decades after initial exposure (24, 38).

### Physical Examination

Among patients with prolonged, persistent symptoms in Lyme disease, the physical examination is often largely normal, and an important initial focus should be to exclude the presence of findings which would suggest another potential cause of the patients' symptoms. Special attention should be paid to the musculoskeletal examination for objective evidence of joint inflammation and swelling. Patients with persistent symptoms after treatment of early localized or early disseminated Lyme disease often have joint pain (i.e., arthralgia) but almost never have inflammatory arthritis with joint inflammation and swelling (33). Oligoarthritis with obvious swelling, especially of the knee, may suggest a site of ongoing infection. Arthrocentesis may then be performed, and the synovial fluid tested by PCR, to evaluate for *B. burgdorferi* infection and the need for further antibiotic therapy. Patients with previously treated late Lyme arthritis however, may have persistent swelling as a manifestation of antibiotic refractory late Lyme arthritis (now also called post-infectious Lyme arthritis), a persistent form of synovitis following antibiotic treatment (53, 94–96). Patients with persistent symptoms who develop polyarthritis with an abnormal joint exam suggestive of rheumatoid arthritis or psoriatic arthritis following treatment for acute Lyme disease have also been described (97). In this case, it is hypothesized that Lyme disease triggered the polyarthritis in susceptible individuals, such as those with a history of psoriasis, but that it is not due to active infection of the joint. Therefore, the aim of treatment should be to control the potentially joint-damaging inflammation using established therapies for managing rheumatoid arthritis or psoriatic arthritis (97).

After treatment for acute neurologic disease such as seventh nerve palsy or radiculititis, the neurologic examination may identify residual deficits, such as cranial nerve damage or radicular pain. These findings may resolve gradually and/or leave residual fixed deficits which may be more or less apparent to the patient from that point on (98). Neurologic findings among patients with persistent symptoms are different however, as they are often more subtle and relate more to symptoms and signs of encephalopathy, and may require consultation for uncertain cases. In patients with untreated late Lyme encephalopathy or persistent symptoms with neurologic manifestations, the most common physical findings are related to memory loss or difficulty finding words, which may be documented on neurocognitive testing (see section Neurocognitive Testing). Hyperreflexia and evidence of upper motor neuron weakness is rarely found (99).

After treatment of late Lyme encephalopathy, more than half of patients will improve. In one study 22% improved but then relapsed with what would now be considered PTLD, with symptoms and signs of encephalopathy (99). Antibiotic therapy is typically effective in resolving obvious signs of vertigo, dizziness, and hearing loss found in neuroborreliosis, and persistent balance instability responds well to vestibular rehabilitation (100). Persistent audiologic complaints were found in a small study of 18 patients with PTLD, and 44% had one or more abnormal pure tone threshold, 31% had abnormally reduced loudness discomfort level, and 17% had abnormal acoustic reflexes at one or more frequencies (101).

In untreated patients with persistent sensory or motor symptoms, examination of the peripheral nerves may demonstrate evidence of peripheral neuropathy, which should be confirmed on electromyography or nerve conduction studies (102). In our clinical case series of patients with PTLD, the most common neurologic exam finding was abnormal vibratory sensation. Thirty-two percent were found to be below age-adjusted threshold values for vibratory sense on either upper or lower extremities using a Rydel-Seiffer 64 Hz tuning fork compared to an estimated, expected 5% in the general population (60, 103). Furthermore, although numbness, tingling, paresthesia, and altered temperature perception are common persistent symptoms and often occur in the context of an otherwise normal physical examination, they should prompt consideration of a possible small fiber neuropathy. This may be pursued through a specialized skin biopsy to measure small nerve fiber density, which may also show evidence of autonomic nervous system involvement as well (104). Development of overt postural orthostatic tachycardia syndrome in PTLD is rare but has been described in the literature (84, 105). Tilt table testing for evidence of orthostatic intolerance syndromes may be useful in order to guide specific interventions.

### Laboratory and Imaging

Patients with persistent symptoms in the context of Lyme disease should undergo blood work at their initial evaluation, which may include a complete blood count, metabolic panel, thyroid testing, erythrocyte sedimentation rate, and C-reactive protein. This is important in order to rule out other symptom causes such as severe anemia, liver, kidney or other metabolic conditions such as diabetes, or other inflammatory or neoplastic conditions. Mild elevations in C-reactive protein have been reported in PTLD (106), however moderate to severe elevations in the erythrocyte sedimentation rate or C-reactive protein are distinctly unusual and should prompt evaluation of another infectious, neoplastic, or autoimmune condition, such as polymyalgia rheumatica.

Two-tier testing was developed for surveillance purposes and should not be used alone outside of clinical judgment in diagnosing and treating Lyme disease (107). Similarly, it is neither sensitive nor specific in clinically evaluating persistent symptoms in the context of previous antibiotic treatment. A positive serology is not required as part of the proposed research case definition for PTLD (14), as antibody levels have not been found to be associated with specific clinical outcomes following treatment for Lyme disease (38, 108). Particularly for those diagnosed early in infection with localized disease, patients may be seronegative on acute testing and it is known that antibiotic treatment appears to blunt the development of a later serologic response on convalescent testing (13). Conversely, patients may be seropositive for both immunoglobulin M or G antibody responses years or decades later after resolution of their infection (109). This may lead to misattribution of current symptoms to Lyme disease, and other causes must always be considered and excluded even in the context of a positive antibody test. Furthermore, two-tier or C6 antibody testing cannot be used as a test of microbiologic cure, which may be of particular concern when treating later stages of the infection, or when infection involves the central nervous system, where antibiotic penetration may be suboptimal (110).

While patients with persistent symptoms who have not been specifically treated for Lyme disease would be expected to have a positive serologic response, this is not always observed clinically. Several factors may account for this, including unintended prior antibiotic exposure for an alternative co-morbid or misdiagnosed condition. Alternatively, patients may fail to meet the exact cut-off criteria despite evidence of some antibody response to *B. burgdorferi*. In these patients, with the exception of synovial fluid PCR to confirm the diagnosis of late Lyme arthritis, direct diagnostic tests such as bacterial culture or PCR of the blood or cerebrospinal fluid (CSF) are often either insensitive or unavailable in non-research settings (111). Culture of *B. burgdorferi* remains a challenge in both untreated and treated patients, and it is uncertain whether persister organisms can be cultured at all, as evidenced by animal models (112).

Serologic testing for other infectious agents in patients who are *B. burgdorferi* seronegative or who remain ill after initial treatment for Lyme disease may be indicated in certain circumstances. For instance, in patients with suggestive presentations or risk factors for specific animal exposures, testing for Brucellosis, Q fever, or Bartonellosis may be indicated. Scientific knowledge of the frequency and relevance of exposure to multiple co-infectious agents, such as *Anaplasma, Ehrlichia*, and *Bartonella* species of bacteria, *Babesia* parasites, or other *Borrelia* species such as *B. miyamotoi*, in persistent symptoms is limited. Symptomatic co-infection of *B. burgdorferi* and *Babesia microti* is well-documented, and may result in chronic illness, especially in patients with an impaired immune system (113). The role of other *Babesia* species, such as *B. duncani*, in persistent symptoms is unknown (114). Infection with *Bartonella* species of bacteria, which can also cause chronic illness, is thought to result primarily from flea bites, although transmission via ticks is an area of emerging knowledge (115). Finally, there has been speculation that non-vector borne infections such as mycoplasma and Epstein-Barr virus may be involved in the perpetuation of chronic symptoms in patients with Lyme disease (116). It should be noted that a positive serologic test for many infections does not equate to on-going infection, as immunological memory can create long-lasting and even life-long antibodies after active infection is resolved.

In patients with untreated late Lyme encephalopathy or persistent symptoms with neurologic manifestations, lumbar puncture may be employed. In these circumstances, an abnormal CSF warrants neurologic consultation and the potential treatment of neuroborreliosis, depending on the clinical circumstances (117). Central nervous system imaging in patients with late Lyme neuroborreliosis and/or neurologic symptoms has not been definitively characterized and more research is warranted. In patients with late Lyme neuroborreliosis, earlier reports of MRI imaging often showed non-specific white matter lesions (118, 119). However, there are currently no imaging findings considered specific for Lyme neuroborreliosis, and significant overlap exists with other neurologic conditions, particularly multiple sclerosis (120). Patients with persistent symptoms, including those with PTLD, have also been studied using various neuroimaging modalities including MRI (121, 122), SPECT (123, 124), and PET (125) scanning techniques. With the exception of one study (121), all identified abnormalities in a subset of individuals within their respective samples. However, imaging studies in Lyme disease have more recently been called into question as a result of advances in imaging technology, knowledge of age-related white matter changes and potential overlap with the general population, and/or increased specificity of diagnostic criteria (126). Newer research techniques, such as those to image central nervous system inflammation using novel PET imaging (127), may aid diagnosis in the future and provide additional insight into the pathophysiology of persistent symptoms.

### Neurocognitive Testing

Several studies have characterized the neurocognitive testing profile of patients with PTLD (32, 128–135), who often complain of memory, focus, concentration, and processing speed difficulties (Figure 1). We recently reported that among patients with PTLD who gave adequate test engagement on validity testing, 7% were found to have cognitive impairment using stringent measures relative to population norms. However, when compared instead to education-based estimates of their own pre-morbid functioning, 34% of the sample showed decline (128). Patients with a history of treated Lyme disease (32), and in particular the subset who report persistent symptoms (128–130, 133) often have specific, modest deficits in verbal memory as a group relative to controls. In one study, these objective deficits were found to be present only in the subset of PTLD patients with abnormal CSF findings, suggesting a neurological basis (133). It has also been noted that patients with PTLD can have deficits in mental activation or information processing speed when initiating a cognitive process, independent of sensory, perceptual, or motor deficits (131). Although mood-related symptoms are often present to a greater degree among patients with PTLD compared to controls (Figure 1), depression has not been shown to be associated with performance on memory testing in this population (129). Moreover, patients with PTLDS appear to have more pronounced problems on memory-related tasks when compared to patients with major depressive disorder (132).

## Etiology

**Figure 2:**
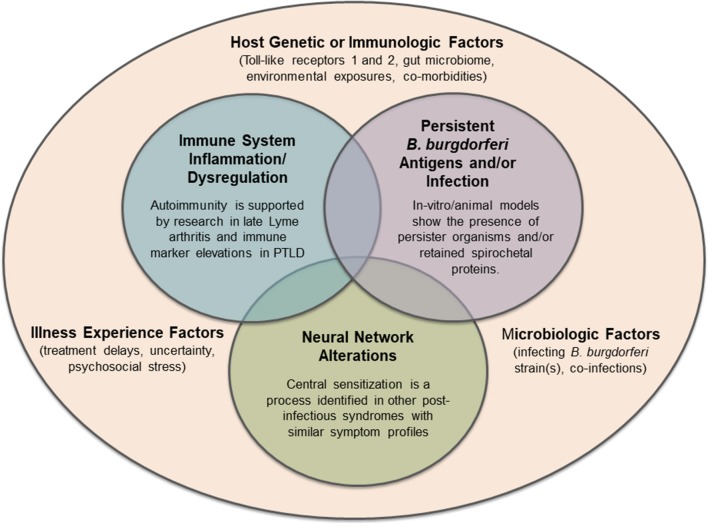
Hypotheses regarding potential mechanisms of persistent symptoms in Lyme disease, all of which may exist within the complex microbiologic, immunologic, genetic, and lived experience of individual patients. Future mechanisms of disease or other pre-disposing factors may still be identified.

*B. burgdorferi* is a zoonosis which has adapted to living in a mammalian host. In its natural reservoir host, the white-footed mouse, it does not appear to cause symptomatic disease. The genome of *B. burgdorferi* does not appear to code for any known toxins, and as such, it does not have the ability to directly damage host tissue (49). Therefore, the symptoms of Lyme disease can be considered mostly due to the host innate and adaptive immune response to infection. For example, early Lyme disease is characterized by high levels of many immune mediators, which may be beneficial in clearing the infection but also can cause symptoms such as fever and malaise (136). In patients with prolonged, persistent symptoms, the host immune response may become dysregulated through inflammatory or secondary autoimmune pathways, or non-specific immune activation. Other systems, such as central neural pathways and networks, may also be disrupted and have a significant impact on symptoms. The primary driver of this initial dysregulation remains unknown, and it may be dependent on or independent of microbial persistence. It is likely that a variety of factors, many of them overlapping and interacting, contribute to this symptom profile (Figure 2). All these illness mechanisms occur in the context of the host genetic and environmental background, variability in the infecting organism, and the illness experience of the patient.

*In vitro* research shows that *B. burgdorferi*, like many bacteria, can form persister organisms either under antibiotic pressure or during stationary phase growth, and that these persister organisms are antibiotic-tolerant and are less likely to be killed with standard antibiotics (137, 138). The fact that symptoms can persist in previously treated patients, including those with PTLD, does not exclude microbial persistence as a hypothesized etiology of the symptoms; moreover, exposure of *B. burgdorferi* to antibiotics is the basis for *in vitro* models of bacterial persistence (138). It remains unknown therefore, whether standard antibiotic therapy may result in partial treatment or generation of persister organisms that may be involved in the ongoing pathophysiology of persistent symptoms. Mouse, dog, and non-human primate model work show evidence of *B. burgdorferi* persistence by tissue histopathology and PCR in antibiotic treated animals (139–142). These persister organisms have been shown to be metabolically active in RNAseq studies and xenodiagnosis with ticks has demonstrated *B. burgdorferi* transmission from antibiotic-treated animals to immunodeficient mice (112). However, they are not easily cultivatable and Koch's postulates have been difficult to prove (140). An alternate explanation for ongoing inflammation is antigen persistence after complete killing of replicating bacteria. In the mouse model, extensive bacterial debris can be visualized after antibiotic treatment despite eradication of cultivatable organisms (143). These antigens may have the ability to specifically activate host immune cells directly or via non-specific bystander activation pathways (144).

Alternatively, antibiotic-refractory late Lyme arthritis, the most studied post-treatment manifestation of Lyme disease, is thought to be autoimmune in nature, as *B. burgdorferi* can no longer be found in the joint or surrounding tissue in patients who have been thoroughly treated (145). Among patients with this form of inflammatory arthritis, high levels of Th17-associated mediators have been found to correlate strongly with autoantibodies to several Lyme disease-specific autoantigens (63). This suggests that immune dysregulation, in the form of a shift toward autoimmune responses, may contribute to on-going synovitis following antibiotic treatment in the joint (146). Investigators have also shown peptidoglycan persistence in synovial fluid of these patients despite the lack of morphologically intact bacteria (147). It is unclear whether this on-going inflammation is sustained by persistent microbial antigens (148), or even the biologic feasibility of chronic antigen persistence in sequestered sites such as the joint.

Investigation into the role of an ongoing immune response in the symptomatology of patients with persistent symptoms is in its earliest stages. A handful of studies have suggested that inflammatory markers such as C-reactive protein, as well as immune mediators such as CCL19 and IL-23, remain elevated for months after completion of antibiotic therapy among patients with persistent symptoms (47, 48, 106). Anti-neural antibody reactivity is higher in those with persistent symptoms, even among those who are seronegative, compared to those who returned to health after treatment for Lyme disease (149). Another study among post-treatment patients with objective memory impairment found a unique proteomic signature in the CSF with specific differentially increased complement cascade proteins (150).

Finally, infection-triggered, post-infectious syndromes have been described for a variety of viruses (151). Given the clinical similarities between many of these syndromes and persistent symptoms in Lyme disease, including fibromyalgia and chronic fatigue syndrome, it has also been hypothesized that analogous underlying mechanisms may contribute to the symptom profile for all of these conditions (152). Specifically, central sensitization is a process of hyperactivation in the central neural pathways, leading to a more intense response to sensory stimuli, which is experienced as hyperalgesia and/or allodynia (152). The presence of depression or anxiety may be related to these altered neural networks and their associated neurotransmitter changes, or it may also evolve under the patient's lived chronic illness experience, which is often marked by uncertainty and newfound significant functional limitations (153). Although the role of central sensitization in fibromyalgia and other syndromes has been previously appreciated, very little research in this area has been conducted among patients with persistent symptoms in Lyme disease.

## Treatment

There are currently no FDA-approved or commonly agreed-upon treatments for patients who have undergone a recommended course of antibiotics for Lyme disease but who continue to have persistent symptoms. Until the pathophysiology of these persistent symptoms is identified and/or a biomarker is developed, it is likely that treatment recommendations will continue to be without consensus. A small number of double-blind, placebo-controlled clinical trials, with a degree of variability in enrollment criteria, intervention, and outcome measures, have been conducted to test whether additional antibiotics are effective (80, 83, 135, 154, 155). One recent study also tested the added benefit of longer-term compared to shorter term antibiotic re-treatment in PTLD (156). In sum, although the study design and interpretation of clinical relevance in the findings of these studies have been debated (157, 158), they have not provided convincing enough evidence of a significant, sustained treatment effect for the Infectious Diseases Society of America (IDSA) to recommend additional antibiotics in their guidelines (14). Furthermore, anecdotal reports of adverse events or even death (159, 160), and the risk of antibiotic resistance at the population level with long-term, untargeted use of antibiotics are often cited as significant concerns (161). By contrast, the International Lyme and Associated Diseases Society (ILADS) have issued markedly different clinical recommendations focused on often open-ended antibiotic treatment of persistent infection and the potential for multiple tick-borne co-infecting agents (162). These recommendations are based on extensive review of the literature supporting the hypothesis of microbial persistence as a mechanism of persistent symptoms in both untreated and previously treated Lyme disease (163). The debate over appropriate and effective treatment strategies for patients with persistent symptoms is one of the primary drivers of the on-going controversy in Lyme disease.

Aside from antibiotics, additional pharmacologic (albeit off-label) or non-pharmacologic therapies for clinical care focus on managing individual symptoms and restoring or improving functioning. For example, pregabalin, and duloxetine may provide some symptom improvement for patients who also meet criteria for fibromyalgia. Tricyclic antidepressants such as nortriptyline are often used for symptomatic management of pain and sleep, and selective serotonin reuptake inhibitors may be indicated for management of secondary depression or anxiety. Other medications for fatigue, such as modafinil, may also be considered but none of these interventions have been subjected to controlled trials (164). Non-pharmacologic interventions such as cognitive-behavioral or other types of therapy as a means to ease symptom burden and help manage the stress of living with a chronic illness may be useful as well. Mindfulness-based stress reduction has been tested among patients with fibromyalgia and shows promise in reducing both symptoms and stress levels (165). A supervised resistance exercise program was shown to increase the number of days feeling healthy and energetic among a small sample of patients with persistent symptoms of Lyme disease (166). The use of complementary, alternative therapies such as essential oils may be promising, however additional *in vivo* work is needed to address safety and pharmacokinetic properties (167).

Additional treatment trials are needed to test the effectiveness of new therapeutic approaches for patients with prolonged, persistent symptoms following recommended treatment for Lyme disease. Large drug-screening efforts have identified new potential antibiotic and non-antibiotic therapeutic targets with activity against *B. burgdorferi* (168–170). Although it is unclear how the data will translate to human disease, *in vitro* and animal models support therapeutics which target persister organisms (137, 171). Furthermore, refinement of treatment protocols for antibiotic-refractory late Lyme arthritis have led to a combination of multiple initial, defined courses of antibiotics followed by a transition to anti-inflammatory therapy when the evidence suggests that infection has been eradicated (53). This may provide a model for future testing of anecdotally suggested, defined, retreatment protocols, particularly among patients with risk factors for persistent symptoms, and conceivably with oral antibiotics which have both anti-infective and anti-inflammatory properties (172).

## Defining Patient Subgroups: Post-Treatment Lyme Disease in the Context of Chronic Lyme Disease

Patients with persistent symptoms related to Lyme disease likely represent a heterogeneous population, which includes previously untreated patients, as well as those treated patients who remain symptomatic. As a result, some (largely those with prior treatment) will manifest primarily patient-reported symptoms while others (largely untreated patients) will present with symptoms in conjunction with objective, physical findings. This heterogeneity is further complicated by variation in terminology and the definitions used by different groups in the field.

Patients with untreated Lyme disease have a significant chance of developing persistent signs and symptoms, primarily in the form of arthritis and less commonly, neurologic disease (2). The best studied and agreed-upon persistent manifestations of untreated Lyme disease are late Lyme arthritis, which may present with joint pain, synovitis, and swelling months to years after initial infection, and its post-treatment sequelae antibiotic-refractory late Lyme arthritis, which may occur in ~10% of patients (173). These manifestations present with objective joint swelling and the presence of joint fluid which can be analyzed by PCR for *B. burgdorferi*, rendering the diagnosis and biologic evaluation of these conditions possible. Similarly, patients with untreated neurologic disease may develop Lyme encephalopathy, manifesting primarily as memory or other cognitive problems. This symptom complex may require further neurocognitive testing, central nervous system imaging, or CSF analyses for evidence of ongoing infection.

**Figure 3:**
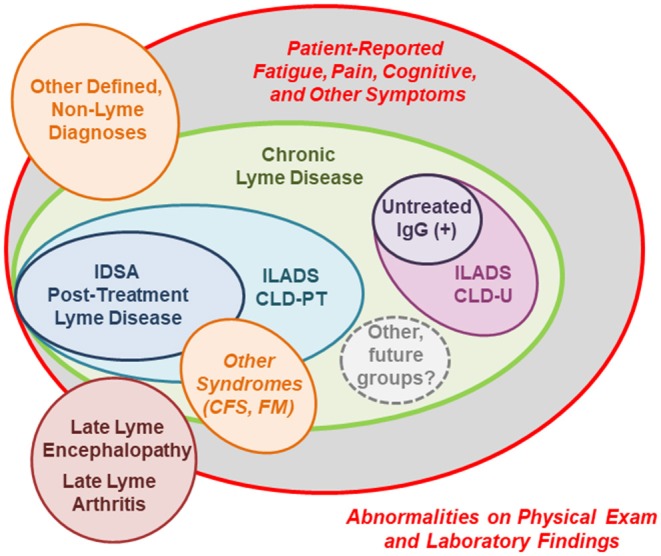
A schematic of clinical- and research-defined patient subgroups among those with persistent symptoms associated with Lyme disease (14, 163, 174). The size of each patient subgroup is not meant to represent actual population frequency, as prevalence data is extremely limited. IDSA, Infectious Diseases Society of America; ILADS, International Lyme and Associated Diseases Society; CLD-PT, Chronic Lyme Disease-Previously Treated; CLD-U, Chronic Lyme Disease-Untreated; IgG, Immunoglobulin G; CFS, Chronic Fatigue Syndrome; FM, Fibromyalgia.

By contrast, the majority of patients with persistent symptoms have primarily patient-reported, non-specific symptoms in the absence of classic physical findings of organ-based damage or disease, and therefore it is often presumed that the source of their illness is unrelated to *B. burgdorferi* exposure (59). In 2007, Feder et al. introduced the concept of multiple sub-categories of patients under the umbrella term “chronic Lyme disease” (CLD) to characterize these patients (174). CLD is a polarizing diagnosis in clinical medicine, with widely divergent definitions and understandings of disease mechanism and effective treatments (175). One key component of the controversy relates to whether CLD is a real disease which is associated pathophysiologically to past or present *B. burgdorferi* infection. We have modified the initial classifications detailed by Feder et al. in the model presented in Figure 3, which depicts the subgroups described in this section. These subgroups are primarily distinguished by the strength of the evidence in their past medical history and in their current clinical presentation for exposure to *B. burgdorferi* (174). It is likely that additional, future sub-groups will continue to be identified as our understanding increases of both the pathophysiology of CLD and the diversity of potential infecting tick-borne pathogens.

Patients with PTLD are the most-studied subgroup of those with CLD, as they represent a narrow, highly specific subset of the broader population of patients with persistent symptoms (14, 174). By definition, these patients had documented Lyme disease prior to developing their chronic symptoms which appeared in spite of recommended antibiotic treatment. In 2006, the IDSA included a proposed case definition for PTLD which stipulates the following key elements: (a) a prior documented episode of Lyme disease meeting CDC criteria in which all objective signs resolve; (b) fatigue, widespread musculoskeletal pain, and/or cognitive difficulties which begin within 6 months, last for at least 6 months, and are significant enough to impair daily function; and (c) the absence of specific co-morbid or pre-existing conditions (outlined in the definition) which could otherwise explain symptoms (14). Notably, PTLD is a mechanistically neutral research definition and the term “post-treatment” refers to the patient's status of having been previously treated with appropriate antibiotics. In our experience, patients who meet this case definition are not uncommon in a Lyme disease referral practice in an endemic area, and represent ~15% of patients referred for evaluation (11, 60).

The aim of this definition was to provide a framework for research, and to limit clinical heterogeneity in study populations (14). In research settings where reliability is methodologically important in order to advance scientific understanding, it offers a way to identify a subset of those patients with on-going symptoms linked temporally to strong evidence of prior exposure to *B. burgdorferi* using the only tools currently available. As such, it is the most accepted and agreed-upon research subgroup of patients, despite having a symptom-defined clinical phenotype which lacks characteristic objective findings. While there are advantages to standardizing a research definition in this way, there are also limitations to the application of this definition in the clinical setting, as there are multiple pathways through which patients who may be suffering from on-going symptoms from Lyme disease may not meet these narrow criteria.

For example, a subset of these patients may have experienced initial delays in diagnosis, and/or misdiagnoses of their presenting signs or symptoms (43, 71, 176–179). The particular clinical difficulties in diagnosing EM (180–182), and/or a lack of EM or acute symptoms, remain on-going issues in community practice and can lead to significant delays in diagnosis and initiation of appropriate treatment. These scenarios have several important implications, as they not only increase risk for later persistent symptoms but they may also set patients on a clinical trajectory that never resembles the classic, textbook manifestations of Lyme disease. Furthermore, by the time patients have on-going, subacute symptoms, misdiagnosis or delays in diagnosis also increase the likelihood that patients will have been exposed to non-ideal antibiotics (43, 176). Partial treatment with non- or minimally-effective antibiotic regimens can explain the high rate of seronegativity and lack of seroconversion in this group. All of these factors hinder or obscure documentation of an initial episode in the medical record which meets CDC criteria for Lyme disease. Lastly, while the exclusion of patients with specific co-morbid conditions creates a high degree of illness specificity, it does not preclude the clinical reality that Lyme disease and its associated persistent sequelae often occur in the context of other pre-existing conditions.

Notably, the ILADS organization has also released a broader, more inclusive definition for CLD which encompasses those patients meeting the IDSA definition for PTLDS, as well as those with similar symptoms who would be excluded for lack of functional impairment or weaker evidence for prior Lyme disease (163). The ILADS CLD definition also differs from the IDSA's PTLDS definition in that it distinguishes between previously treated (CLD-PT) and untreated (CLD-U) presentations, and specifies on-going *B. burgdorferi sensu lato* infection as the underlying illness mechanism for both. However, ILADS CLD is a symptom-based definition and therefore ongoing infection is difficult to assess in individual patients with no currently available biomarker test, and given the large degree of symptom overlap between CLD and other illnesses.

Another unique subgroup of patients are those with only persistent, patient-reported symptoms similar to those reported in PTLD, a positive immunoglobulin G antibody response supporting prior exposure to *B. burgdorferi*, and no history of treatment for Lyme disease. Patients with this clinical presentation represented ~6% of those referred for possible Lyme disease in our retrospective chart review (11). As these patients lack classic, objective signs of Lyme disease, they are often characterized as having ambiguous evidence of *B. burgdorferi* infection (174). However, this group may represent an interesting, intermediate clinical phenotype between archetypal Lyme disease and PTLD, as these types of presentations may occur before the onset of late Lyme arthritis, during which time objective manifestations may be transient (10).

Finally, given the lack of an available biomarker, there will always be a subset of patients with missed diagnoses of other diseases and conditions with similar symptoms, who initially seek evaluation for CLD with non-specific symptoms (11, 90, 91). This may include patients with other defined metabolic, inflammatory, neoplastic, or infectious diseases which can be differentiated by laboratory testing. It may also include patients who meet clinical criteria for syndromes such as fibromyalgia or chronic fatigue syndrome, which also have a high burden of non-specific symptoms such as fatigue and pain and lack clinically available biomarkers. These syndromes are commonly diagnosed, depending on the type and geographic location of the practice, in referral clinics among patients both with and without evidence for prior Lyme disease in their history (90, 91). Similarly, persistent symptoms in Lyme disease are sometimes attributed to misdiagnoses of common, chronic co-morbidities. Although this scenario does present an added degree of clinical complexity, it is important to consider the possible co-occurrence of two disease processes and the role of interaction between the two.

## Current Challenges and Future Priorities

Patients with PTLD represent a substantial burden to the United States health care system. In a large, health insurance claims analysis of 47 million members, estimated total direct medical costs from Lyme disease were between $712 million and $1.3 billion per year, with a significant portion of these specifically due to PTLD-related costs (183). The same study found that the adjusted odds of any PTLD-related symptom diagnosis following Lyme disease was 4.77 higher than age-, sex-, enrollment year-, region- and payer type-matched controls without Lyme disease, and that those patients with Lyme disease who went on to have at least one PTLD symptom had over twice the average total health care costs as those who did not (183). These cost estimates do not reflect additional indirect, non-medical, and lost productivity costs to patients, which may be substantial in a population with a chronic and significant illness impact on quality of life (80, 153, 184). Novel preventative approaches to reduce incidence of new Lyme disease cases, as well as physician and community educational interventions to increase awareness and reduce diagnostic delays and misdiagnosis, are needed to reduce costs and improve patient outcomes.

In a 2010 survey of primary care physicians in Connecticut, 50% responded that the did not “believe” in CLD, however almost all of the remainder (48%) responded that they were undecided or unsure (185). Despite the impression given that very few reputable physicians “believe” in CLD, many physicians and public health faculty acknowledge a real problem that is not just being driven by a small group of patients, physicians and advocacy groups (186). Given the wide variety of prescription, over the counter, and alternative treatments still currently being prescribed by physicians or sought independently by patients, there is a need to rigorously test new evidence-based therapeutic options for patients with persistent symptoms (187). Physicians would be aided greatly by illness biomarkers and effective tests of cure, as two-tier serology alone cannot be used in the post-treatment period for this purpose. However, such progress rests upon basic and translational scientific advancements in elucidating the biologic mechanisms of disease in this population.

Finally, only a handful of qualitative studies have been conducted which address the lived experience of patients with persistent symptoms (42, 153, 188–190). However, it is evident from this small body of literature that a key component of this experience is an often complex and protracted interaction with the health care system. Notably, patient frustration, financial burden, and “a long road to diagnosis” (189) are characteristic in the community practice of medicine (188). These factors are compounded by an immediate need to re-negotiate physical and social identities to the “new normal” of chronic illness, often without the same level of medical support and certainty afforded patients with non-contested conditions (153). We hypothesize that increased validation of the illness experience will improve interactions with the health care system and may also have under-appreciated downstream effects on patients' quality of life, coping, resilience, and even the physical burden of disease (191). An understanding of the ways in which the historical social construction of this illness and interactions with the health care system itself may contribute independently of, but in parallel to, biologic disease processes, is a final, important component of a multidisciplinary approach to alleviate patient suffering (192).

## Conclusions

It is likely that the number of new Lyme disease cases will continue to increase in the coming decades, and consequently so will the number of patients with the variety of clinical presentations described in this article as having persistent symptoms. These symptoms are significant for the magnitude of their impact on the broader health care system, as well as on the quality of life of individual affected patients. Although much progress has been made to characterize and understand the most common manifestations of Lyme disease in the almost 50 years since it was first identified, many fundamental questions surrounding persistent symptoms remain unanswered. Beyond the uncertainty and the controversy, real opportunity exists for scientific insight into not just Lyme disease but also the increasing numbers of patients with unexplained symptoms and syndromes for which modern medicine does not currently offer explanation or treatment.

The identification of patient subgroups is an important way to address these questions and understand the heterogeneity of this patient population. PTLD is one such defined sequelae of Lyme disease which exists within the broader universe of patients with persistent symptoms. Irrespective of the underlying pathophysiology of the illness, it is a useful tool which can be operationalized in research settings where specificity and standardization is essential. Importantly, it can be used as a starting point to move the field forward scientifically, and eventually to understand other clinical subgroups where the established link to prior Lyme disease may exist but is less firm. Despite significant challenges, there is a critical need to develop and refine scientifically rigorous, multidisciplinary means of engaging with these more complex and controversial presentations of Lyme disease.

## Author Contributions

AR and JA contributed to the organization and design of the manuscript, performed literature reviews, participated in drafting sections of the manuscript, and in revising, reading, and approving the final submitted version.

### Conflict of Interest

JA has been granted a United States patent on the use of the T-cell chemokine CCL19 to identify patients at risk for PTLD who may potentially benefit from further treatment. The remaining author declares that the research was conducted in the absence of any commercial or financial relationships that could be construed as a potential conflict of interest.

## References

[1] Mead PS. Epidemiology of Lyme disease. Infect Dis Clin North Am. (2015) 29:187–210. 10.1016/j.idc.2015.02.010 25999219

[2] Steere AC Strle F Wormser GP Hu LT Branda JA Hovius JW. Lyme borreliosis. Nat Rev Dis Prim. (2016) 2:16090. 10.1038/nrdp.2016.90 27976670 PMC5539539

[3] Hinckley AF Connally NP Meek JI Johnson BJ Kemperman MM Feldman KA. Lyme disease testing by large commercial laboratories in the United States. Clin Infect Dis. (2014) 59:676–81. 10.1093/cid/ciu397 24879782 PMC4646413

[4] Ogden NH Radojevic M Wu X Duvvuri VR Leighton PA Wu J. Estimated effects of projected climate change on the basic reproductive number of the Lyme disease vector Ixodes scapularis. Env Heal Perspect. (2014) 122:631–8. 10.1289/ehp.1307799 24627295 PMC4050516

[5] Stone BL Tourand Y Brissette CA. Brave new worlds: the expanding universe of Lyme disease. Vector-Borne Zoonotic Dis. (2017) 17:619–29. 10.1089/vbz.2017.2127 28727515 PMC5576071

[6] Gasmi S Ogden N Lindsay L Burns S Fleming S Badcock J. Surveillance for Lyme disease in Canada: 2009–2015. Canada Commun Dis Rep. (2017) 43:194–9. 10.14745/ccdr.v43i10a01 29770045 PMC5764744

[7] Nelder M Wijayasri S Russell C Johnson K Marchand-Austin A Cronin K. The continued rise of Lyme disease in Ontario, Canada: 2017. Canada Commun Dis Rep. (2018) 44:231–6. 10.14745/ccdr.v44i10a01 31524884 PMC6707478

[8] Wormser GP. Hematogenous dissemination in early Lyme disease. Wien Klin Wochenschr. (2006) 118:634–7. 10.1007/s00508-006-0688-9 17160600

[9] Steere AC Malawista SE Snydman DR Shope RE Andiman WA Ross MR. Lyme arthritis: an epidemic of oligoarticular arthritis in children and adults in three connecticut communities. Arthritis Rheum. (1977) 20:7–17. 10.1002/art.1780200102 836338

[10] Steere AC Schoen RT Taylor E. The clinical evolution of Lyme arthritis. Ann Intern Med. (1987) 107:725–31. 10.7326/0003-4819-107-5-725 3662285

[11] Aucott JN Seifter A Rebman AW. Probable late Lyme disease: a variant manifestation of untreated *Borrelia burgdorferi* infection. BMC Infect Dis. (2012) 12:173. 10.1186/1471-2334-12-173 22853630 PMC3449205

[12] Moore A Nelson C Molins C Mead P Schriefer M. Current guidelines, common clinical pitfalls, and future directions for laboratory diagnosis of Lyme disease, United States. Emerg Infect Dis. (2016) 22:1169–77. 10.3201/2207.151694 27314832 PMC4918152

[13] Aguero-Rosenfeld ME Wang G Schwartz I Wormser GP. Diagnosis of Lyme borreliosis. Clin Microbiol Rev. (2005) 18:484–509. 10.1128/CMR.18.3.484-509.2005 16020686 PMC1195970

[14] Wormser GP Dattwyler RJ Shapiro ED Halperin JJ Steere AC Klempner MS. The clinical assessment, treatment, and prevention of Lyme disease, human granulocytic anaplasmosis, and babesiosis: clinical practice guidelines by the Infectious Diseases Society of America. Clin Infect Dis. (2006) 43:1089–134. 10.1086/508667 17029130

[15] Luft BJ Dattwyler RJ Johnson RC Luger SW Bosler EM Rahn DW Masters EJ. Azithromycin compared with amoxicillin in the treatment of erythema migrans. A double-blind, randomized, controlled trial. Ann Intern Med. (1996) 124:785–91. 10.7326/0003-4819-124-9-199605010-00002 8610947

[16] Robert B Lugcr SW Fratik E Wisniewski M Collins JJ Wormser GP Comparison of cefuroxime axetil and doxycycline in the treatment of early Lyme disease. Ann Intern Med. (1992) 117:273–80. 10.7326/0003-4819-117-4-273 1637021

[17] Dattwyler RJ Volkman DJ Conaty SM Platkin SP Luft BJ. Amoxycillin plus probenecid versus doxycycline for treatment of erythema migrans borreliosis. Lancet. (1990) 336:1404–6. 10.1016/0140-6736(90)93103-V 1978873

[18] Dattwyler RJ Wormser GP Rush TJ Finkel MF Schoen RT Grunwaldt E. A comparison of two treatment regimens of ceftriaxone in late Lyme disease. Wien Klin Wochenschr. (2005) 117:393–7. 10.1007/s00508-005-0361-8 16053194

[19] Wormser GP Ramanathan R Nowakowski J McKenna D Holmgren D Visintainer P. Duration of antibiotic therapy for early Lyme disease. A randomized, double-blind, placebo-controlled trial. Ann Intern Med. (2003) 138:697–704. 10.7326/0003-4819-138-9-200305060-00005 12729423

[20] Luger SW Paparone P Wormser GP Nadelman RB Grunwaldt E Gomez G. Comparison of cefuroxime axetil and doxycycline in treatment of patients with early Lyme disease associated with erythema migrans. Antimicrob Agents Chemother. (1995) 39:661–7. 10.1128/AAC.39.3.661 7793869 PMC162601

[21] Dattwyler RJ Luft BJ Kunkel MJ Finkel MF Wormser GP Rush TJ. Ceftriaxone compared with doxycycline for the treatment of acute disseminated Lyme disease. N Engl J Med. (1997) 337:289–94. 10.1056/NEJM199707313370501 9233865

[22] Gerber MA Shapiro ED Burke GS Parcells VJ Bell GL. Lyme disease in children in southeastern Connecticut. N Engl J Med. (1996) 335:1270–4. 10.1056/NEJM199610243351703 8857006

[23] Aucott JN Rebman AW Crowder LA Kortte KB. Post-treatment Lyme disease syndrome symptomatology and the impact on life functioning: is there something here? Qual Life Res. (2013) 22:75–84. 10.1007/s11136-012-0126-6 22294245 PMC3548099

[24] Weitzner E McKenna D Nowakowski J Scavarda C Dornbush R Bittker S. Long-term assessment of post-treatment symptoms in patients with culture-confirmed early Lyme disease. Clin Infect Dis. (2015) 61:1800–6. 10.1093/cid/civ735 26385994 PMC4657537

[25] Ballantyne C The chronic debate over Lyme disease. Nat Med. (2008) 14:1135–9. 10.1038/nm1108-1135 18989271

[26] Davis G Nichter M The Lyme wars: the effects of biocommunicability, gender, and epistemic politics on health activation and Lyme science. In: Smith-Morris C, editor. Diagnostic Controversy: Cultural Perspectives on Competing Knowledge in Healthcare. New York, NY: Routledge (2015). p. 215–46.

[27] Stricker RB Johnson L Chronic Lyme disease and the “axis of evil.” Futur Microbiol. (2008) 3:621–4. 10.2217/17460913.3.6.621 19072179

[28] Lantos PM. Chronic Lyme disease: the controversies and the science. Expert Rev Anti Infect Ther. (2011) 9:787–97. 10.1586/eri.11.63 21810051

[29] Steere AC Hutchinson GJ Rahn DW Sigal LH Craft JE DeSanna ET. Treatment of the early manifestations of Lyme disease. Ann Intern Med. (1983) 99:22–6. 10.7326/0003-4819-99-1-22 6407378

[30] Dattwyler RJ Halperin JJ. Failure of tetracycline therapy in early Lyme disease. Arthritis Rheum. (1987) 30:448–50. 10.1002/art.1780300414 3580012

[31] Asch ES Bujak DI Weiss M Peterson MG Weinstein A. Lyme disease: an infectious and postinfectious syndrome. J Rheumatol. (1994) 21:454–461. 8006888

[32] Shadick NA Phillips CB Logigian EL Steere AC Kaplan RF Berardi VP. The long-term clinical outcomes of Lyme disease. A population-based retrospective cohort study. Ann Intern Med. (1994) 121:560–7. 10.7326/0003-4819-121-8-199410150-00002 8085687

[33] Shadick NA Phillips CB Sangha O Logigian EL Kaplan RF Wright EA. Musculoskeletal and neurologic outcomes in patients with previously treated Lyme disease. Ann Intern Med. (1999) 131:919–26. 10.7326/0003-4819-131-12-199912210-00003 10610642

[34] Centers for Disease Control and Prevention Lyme Disease Surveillance and Available Data. Available online at: https://www.cdc.gov/lyme/stats/survfaq.html (accessed December 3, 2019).

[35] Centers for Disease Control and Prevention. Lyme Disease Data and Surveillance. Available online at: https://www.cdc.gov/lyme/datasurveillance/index.html (accessed October 31, 2019).

[36] DeLong A Hsu M Kotsoris H. Estimation of cumulative number of post-treatment Lyme disease cases in the US, 2016 and 2020. BMC Public Health. (2019) 19:352. 10.1186/s12889-019-6681-9 31014314 PMC6480773

[37] Nowakowski J Nadelman RB Sell R McKenna D Cavaliere LF Holmgren D. Long-term follow-up of patients with culture-confirmed Lyme disease. Am J Med. (2003) 115:91–6. 10.1016/S0002-9343(03)00308-5 12893393

[38] Kalish RA Kaplan RF Taylor E Jones-Woodward L Workman K Steere AC. Evaluation of study patients with Lyme disease, 10-20-year follow-up. J Infect Dis. (2001) 183:453–60. 10.1086/318082 11133377

[39] Wills AB Spaulding AB Adjemian J Prevots DR Turk SP Williams C. Long-term follow-up of patients with Lyme disease: longitudinal analysis of clinical and quality-of-life measures. Clin Infect Dis. (2016) 62:1546–51. 10.1093/cid/ciw189 27025825 PMC4885655

[40] Sarfati D Koczwara B Jackson C. The impact of comorbidity on cancer and its treatment. CA Cancer J Clin. (2016) 66:337–50. 10.3322/caac.21342 26891458

[41] Bonsignore MR Baiamonte P Mazzuca E Castrogiovanni A Marrone O. Obstructive sleep apnea and comorbidities: a dangerous liaison. Multidiscip Respir Med. (2019) 14:8. 10.1186/s40248-019-0172-9 30809382 PMC6374907

[42] Hirsch AG Herman RJ Rebman A Moon KA Aucott J Heaney C. Obstacles to diagnosis and treatment of Lyme disease in the USA: a qualitative study. BMJ Open. (2018) 8:e021367. 10.1136/bmjopen-2017-021367 29895655 PMC6009554

[43] Aucott J Morrison C Munoz B Rowe PC Schwarzwalder A West SK. Diagnostic challenges of early Lyme disease: lessons from a community case series. BMC Infect Dis. (2009) 9:79. 10.1186/1471-2334-9-79 19486523 PMC2698836

[44] Jowett N Gaudin RA Banks CA Hadlock TA. Steroid use in Lyme disease-associated facial palsy is associated with worse long-term outcomes. Laryngoscope. (2017) 127:1451–8. 10.1002/lary.26273 27598389

[45] Wormser GP McKenna D Scavarda C Karmen C. Outcome of facial palsy from Lyme disease in prospectively followed patients who had received corticosteroids. Diagn Microbiol Infect Dis. (2018) 91:336–8. 10.1016/j.diagmicrobio.2018.03.016 29720355

[46] Blum LK Adamska JZ Martin DS Rebman AW Elliott SE Cao RRL. Robust B cell responses predict rapid resolution of Lyme disease. Front Immunol. (2018) 9:1634. 10.3389/fimmu.2018.01634 30072990 PMC6060717

[47] Aucott JN Soloski MJ Rebman AW Crowder LA Lahey LJ Wagner CA. CCL19 as a chemokine risk factor for posttreatment Lyme disease syndrome: a prospective clinical cohort study. Clin Vaccine Immunol. (2016) 23:757–66. 10.1128/CVI.00071-16 27358211 PMC5014924

[48] Strle K Stupica D Drouin EE Steere AC Strle F. Elevated levels of IL-23 in a subset of patients with post-Lyme disease symptoms following erythema migrans. Clin Infect Dis. (2014) 58:372–80. 10.1093/cid/cit735 24218102 PMC3890340

[49] Tilly K Rosa PA Stewart PE. Biology of infection with *Borrelia burgdorferi*. Infect Dis Clin North Am. (2008) 22:217–34. 10.1016/j.idc.2007.12.013 18452798 PMC2440571

[50] Crowder CD Matthews HE Schutzer S Rounds MA Luft BJ Nolte O. Genotypic variation and mixtures of Lyme Borrelia in Ixodes ticks from North America and Europe. PLoS ONE. (2010) 5:e10650. 10.1371/journal.pone.0010650 20498837 PMC2871049

[51] Seinost G Dykhuizen DE Dattwyler RJ Golde WT Dunn JJ Wang IN. Four clones of *Borrelia burgdorferi* sensu stricto cause invasive infection in humans. Infect Immun. (1999) 67:3518–24. 10.1128/IAI.67.7.3518-3524.1999 10377134 PMC116539

[52] Wormser GP Brisson D Liveris D Hanincova K Sandigursky S Nowakowski J. *Borrelia burgdorferi* genotype predicts the capacity for hematogenous dissemination during early Lyme disease. J Infect Dis. (2008) 198:1358–64. 10.1086/592279 18781866 PMC2776734

[53] Steere AC Angelis SM. Therapy for Lyme arthritis: strategies for the treatment of antibiotic-refractory arthritis. Arthritis Rheum. (2006) 54:3079–86. 10.1002/art.22131 17009226

[54] Strle K Jones KL Drouin EE Li X Steere AC. *Borrelia burgdorferi* RST1 (OspC type A) genotype is associated with greater inflammation and more severe Lyme disease. Am J Pathol. (2011) 178:2726–39. 10.1016/j.ajpath.2011.02.018 21641395 PMC3123987

[55] Mosel MR Carolan HE Rebman AW Castro S Massire C Ecker DJ Molecular testing of serial blood specimens from patients with early Lyme disease during treatment reveals changing co-infection with mixtures of *Borrelia burgdorferi* genotypes. Antimicrob Agents Chemother. (2019) 63:e00237–19. 10.1128/AAC.00237-19 31036693 PMC6591639

[56] Seinost G Golde WT Berger BW Dunn JJ Qiu D Dunkin DS. Infection with multiple strains of *Borrelia burgdorferi* sensu stricto in patients with Lyme disease. Arch Dermatol. (1999) 135:1329–33. 10.1001/archderm.135.11.1329 10566830

[57] Krause PJ Telford SR Spielman A Sikand V Ryan R Christianson D. Concurrent Lyme disease and babesiosis. Evidence for increased severity and duration of illness. JAMA. (1996) 275:1657–60. 10.1001/jama.1996.03530450047031 8637139

[58] Schwartz AM Hinckley AF Mead PS Hook SA Kugeler KJ. Surveillance for Lyme Disease - United States, 2008-2015. MMWR Surveill Summ. (2017) 66:1–12. 10.15585/mmwr.ss6622a1 29120995 PMC5829628

[59] Weitzner E Visintainer P Wormser GP. Comparison of males versus females with culture-confirmed early Lyme disease at presentation and at 11–20 years after diagnosis. Diagn Microbiol Infect Dis. (2016) 85:493–5. 10.1016/j.diagmicrobio.2016.04.012 27230991

[60] Rebman AW Bechtold KT Yang T Mihm EA Soloski MJ Novak CB. The clinical, symptom, and quality-of-life characterization of a well-defined group of patients with posttreatment Lyme disease syndrome. Front Med. (2017) 4:224. 10.3389/fmed.2017.00224 29312942 PMC5735370

[61] Wormser GP Shapiro ED. Implications of gender in chronic Lyme disease. J Womens Heal. (2009) 18:831–4. 10.1089/jwh.2008.1193 19514824 PMC2913779

[62] Wang TJ Sangha O Phillips CB Wright EA Lew RA Fossel AH. Outcomes of children treated for Lyme disease. J Rheumatol. (1998) 25:2249–53. 9818672

[63] Adams WV Rose CD Eppes SC Klein JD. Long-term cognitive effects of Lyme disease in children. Appl Neuropsychol. (1999) 6:39–45. 10.1207/s15324826an0601_6 10382570

[64] Gerber MA Zemel LS Shapiro ED. Lyme arthritis in children: clinical epidemiology and long-term outcomes. Pediatrics. (1998) 102:905–8. 10.1542/peds.102.4.905 9755263

[65] Weitzner E Visintainer P Wormser GP. Impact of patient age on clinical features, serologic test reactivity and long-term outcome of culture-confirmed early Lyme disease. Diagn Microbiol Infect Dis. (2017) 89:300–2. 10.1016/j.diagmicrobio.2017.09.007 29137719

[66] Moon KA Pollak J Hirsch AG Aucott JN Nordberg C Heaney CD. Epidemiology of Lyme disease in Pennsylvania 2006-2014 using electronic health records. Ticks Tick Borne Dis. (2019) 10:241–50. 10.1016/j.ttbdis.2018.10.010 30420251

[67] Lantos PM. Chronic Lyme disease. Infect Dis Clin North Am. (2015) 29:325–40. 10.1016/j.idc.2015.02.006 25999227 PMC4477530

[68] Halperin JJ. Lyme disease: neurology, neurobiology, and behavior. Clin Infect Dis. (2014) 58:1267–72. 10.1093/cid/ciu106 24571864

[69] Nadelman RB Hanincova K Mukherjee P Liveris D Nowakowski J McKenna D. Differentiation of reinfection from relapse in recurrent Lyme disease. N Engl J Med. (2012) 367:1883–90. 10.1056/NEJMoa1114362 23150958 PMC3526003

[70] Osterhoudt KC Zaoutis T Zorc JJ. Lyme disease masquerading as brown recluse spider bite. Ann Emerg Med. (2002) 39:558–61. 10.1067/mem.2002.119509 11973566

[71] Aucott JN Seifter A. Misdiagnosis of early Lyme disease as the summer flu. Orthop Rev. (2011) 3:e14. 10.4081/or.2011.e14 22053255 PMC3206512

[72] Wessely S. Chronic fatigue: symptom and syndrome. Ann Intern Med. (2001) 134:838–43. 10.7326/0003-4819-134-9_Part_2-200105011-00007 11346319

[73] Murphy KR Han JL Yang S Hussaini SMQ Elsamadicy AA Parente B. Prevalence of specific types of pain diagnoses in a sample of United States adults. Pain Physician. (2017) 20:E257–68. 28158163

[74] Luck T Roehr S Rodriguez FS Schroeter ML Witte AV Hinz A. Memory-related subjective cognitive symptoms in the adult population: prevalence and associated factors - results of the LIFE-Adult-Study. BMC Psychol. (2018) 6:23. 10.1186/s40359-018-0236-1 29784047 PMC5963184

[75] Kaplan RF Meadows ME Vincent LC Logigian EL Steere AC. Memory impairment and depression in patients with Lyme encephalopathy: comparison with fibromyalgia and nonpsychotically depressed patients. Neurology. (1992) 42:1263–7. 10.1212/WNL.42.7.1263 1620329

[76] Fallon J Bujak DI Guardino S Weinstein A. The Fibromyalgia Impact Questionnaire: a useful tool in evaluating patients with post-Lyme disease syndrome. Arthritis Care Res. (1999) 12:42–7. 10513489 10.1002/1529-0131(199902)12:1<42::aid-art7>3.0.co;2-3

[77] Gaudino EA Coyle PK Krupp LB. Post-Lyme syndrome and chronic fatigue syndrome. Neuropsychiatric similarities and differences. Arch Neurol. (1997) 54:1372–6. 10.1001/archneur.1997.00550230045015 9362985

[78] Fallon BA Zubcevik N Bennett C Doshi S Rebman AW Kishon R. The General Symptom Questionnaire-30 (GSQ-30): a brief measure of multi-system symptom burden in Lyme disease. Front Med. (2019) 6:283. 10.3389/fmed.2019.00283 31867334 PMC6908481

[79] Ware JE Kosinski M Dewey JE How to Score Version 2 of the SF-36 Health Survey. Lincoln, RI: Quality Metric Inc (2000). 10.1097/00007632-200012150-00008

[80] Klempner MS Hu LT Evans J Schmid CH Johnson GM Trevino RP. Two controlled trials of antibiotic treatment in patients with persistent symptoms and a history of Lyme disease. N Engl J Med. (2001) 345:85–92. 10.1056/NEJM200107123450202 11450676

[81] Chandra AM Keilp JG Fallon BA. Correlates of perceived health-related quality of life in post-treatment Lyme encephalopathy. Psychosomatics. (2013) 54:552–9. 10.1016/j.psym.2013.04.003 23845316 PMC5507690

[82] Krupp LB LaRocca NG Muir-Nash J Steinberg AD. The fatigue severity scale. Application to patients with multiple sclerosis and systemic lupus erythematosus. Arch Neurol. (1989) 46:1121–3. 10.1001/archneur.1989.00520460115022 2803071

[83] Krupp LB Hyman LG Grimson R Coyle PK Melville P Ahnn S. Study and treatment of post Lyme disease (STOP-LD): a randomized double masked clinical trial. Neurology. (2003) 60:1923–30. 10.1212/01.WNL.0000071227.23769.9E 12821734

[84] Kanjwal K Karabin B Kanjwal Y Grubb BP. Postural orthostatic tachycardia syndrome following Lyme disease. Cardiol J. (2011) 18:63–6. 10.1097/MJT.0b013e3181da0763 21305487

[85] Zimering JH Williams MR Eiras ME Fallon BA Logigian EL Dworkin RH. Acute and chronic pain associated with Lyme borreliosis: clinical characteristics and pathophysiologic mechanisms. Pain. (2014) 155:1435–8. 10.1016/j.pain.2014.04.024 24769365

[86] Cairns V Godwin J. Post-Lyme borreliosis syndrome: a meta-analysis of reported symptoms. Int J Epidemiol. (2005) 34:1340–5. 10.1093/ije/dyi129 16040645

[87] Wormser GP Weitzner E McKenna D Nadelman RB Scavarda C Farber S. Long-term assessment of fibromyalgia in patients with culture-confirmed Lyme disease. Arthritis Rheumatol. (2015) 67:837–9. 10.1002/art.38972 25470117

[88] Lobraico J Butler A Petrini J Ahmadi R. New insights into stages of Lyme disease symptoms from a novel hospital-based registry. J Prim Care Community Heal. (2014) 5:284–7. 10.1177/2150131914540693 24970880

[89] Dinerman H Steere AC. Lyme disease associated with fibromyalgia. Ann Intern Med. (1992) 117:281–5. 10.7326/0003-4819-117-4-281 1637022

[90] Sigal LH. Summary of the first 100 patients seen at a Lyme disease referral center. Am J Med. (1990) 88:577–81. 10.1016/0002-9343(90)90520-N 2346158

[91] Steere AC Taylor E McHugh GL Logigian EL. The overdiagnosis of Lyme disease. JAMA. (1993) 269:1812–6. 10.1001/jama.269.14.1812 8459513

[92] Vazquez M Sparrow SS Shapiro ED. Long-term neuropsychologic and health outcomes of children with facial nerve palsy attributable to Lyme disease. Pediatrics. (2003) 112:e93–7. 10.1542/peds.112.2.e93 12897313

[93] Zomer TP Barendregt JNM van Kooten B van Bemmel T Landman GW van Hees BC. Non-specific symptoms in adult patients referred to a Lyme centre. Clin Microbiol Infect. (2019) 25:67–70. 10.1016/j.cmi.2018.09.016 30287411

[94] Lochhead RB Strle K Kim ND Kohler MJ Arvikar SL Aversa JM. MicroRNA expression shows inflammatory dysregulation and tumor-like proliferative responses in joints of patients with postinfectious Lyme arthritis. Arthritis Rheumatol. (2017) 69:1100–10. 10.1002/art.40039 28076897 PMC5406251

[95] Strle K Sulka KB Pianta A Crowley JT Arvikar SL Anselmo A. T-Helper 17 cell cytokine responses in Lyme disease correlate with *Borrelia burgdorferi* antibodies during early infection and with autoantibodies late in the illness in patients with antibiotic-refractory Lyme arthritis. Clin Infect Dis. (2017) 64:930–8. 10.1093/cid/cix002 28077518 PMC5850331

[96] Pianta A Drouin EE Crowley JT Arvikar S Strle K Costello CE. Annexin A2 is a target of autoimmune T and B cell responses associated with synovial fibroblast proliferation in patients with antibiotic-refractory Lyme arthritis. Clin Immunol. (2015) 160:336–41. 10.1016/j.clim.2015.07.005 26187145 PMC4582008

[97] Arvikar SL Crowley JT Sulka KB Steere AC. Autoimmune arthritides, rheumatoid arthritis, psoriatic arthritis, or peripheral spondyloarthritis following Lyme Disease. Arthritis Rheumatol. (2017) 69:194–202. 10.1002/art.39866 27636905 PMC5195856

[98] Berglund J Stjernberg L Ornstein K Tykesson-Joelsson K Walter H. 5-y Follow-up study of patients with neuroborreliosis. Scand J Infect Dis. (2002) 34:421–5. 10.1080/00365540110080421 12160168

[99] Logigian EL Kaplan RF Steere AC. Chronic neurologic manifestations of Lyme disease. N Engl J Med. (1990) 323:1438–44. 10.1056/NEJM199011223232102 2172819

[100] Jozefowicz-Korczynska M Zamyslowska-Szmytke E Piekarska A Rosiak O. Vertigo and severe balance instability as symptoms of Lyme disease—literature review and case report. Front Neurol. (2019) 10:1172. 10.3389/fneur.2019.01172 31798513 PMC6861545

[101] Shotland LI Mastrioanni MA Choo DL Szymko-Bennett YM Dally LG Pikus AT. Audiologic manifestations of patients with post-treatment Lyme disease syndrome. Ear Hear. (2003) 24:508–17. 10.1097/01.AUD.0000100205.25774.5F 14663350

[102] Logigian EL Steere AC. Clinical and electrophysiologic findings in chronic neuropathy of Lyme disease. Neurology. (1992) 42:303–11. 10.1212/WNL.42.2.303 1310529

[103] Martina IS van Koningsveld R Schmitz PI van der Meché FG van Doorn PA. Measuring vibration threshold with a graduated tuning fork in normal aging and in patients with polyneuropathy. European Inflammatory Neuropathy Cause and Treatment (INCAT) group. J Neurol Neurosurg Psychiatry. (1998) 65:743–7. 10.1136/jnnp.65.5.743 9810949 PMC2170371

[104] Novak P Felsenstein D Mao C Octavien NR Zubcevik N. Association of small fiber neuropathy and post treatment Lyme disease syndrome. PLoS ONE. (2019) 14:e0212222. 10.1371/journal.pone.0212222 30753241 PMC6372188

[105] Noyes AM Kluger J. A tale of two syndromes: Lyme disease preceding postural orthostatic tachycardia syndrome. Ann Noninvasive Electrocardiol. (2015) 20:82–6. 10.1111/anec.12158 24830783 PMC6931862

[106] Uhde M Ajamian M Li X Wormser GP Marques A Alaedini A. Expression of C-reactive protein and serum amyloid A in early to late manifestations of Lyme disease. Clin Infect Dis. (2016) 63:1399–404. 10.1093/cid/ciw599 27585799 PMC5106611

[107] Perea AE Hinckley AF Mead PS. Evaluating the potential misuse of the Lyme disease surveillance case definition. Public Health Rep. (2020) 135:16–7. 10.1177/0033354919890024 31800351 PMC7119247

[108] Fleming RV Marques AR Klempner MS Schmid CH Dally LG Martin DS. Pre-treatment and post-treatment assessment of the C(6) test in patients with persistent symptoms and a history of Lyme borreliosis. Eur J Clin Microbiol Infect Dis. (2004) 23:615–8. 10.1007/s10096-004-1163-z 15243815

[109] Kalish RA McHugh G Granquist J Shea B Ruthazer R Steere AC. Persistence of immunoglobulin M or immunoglobulin G antibody responses to *Borrelia burgdorferi* 10-20 years after active Lyme disease. Clin Infect Dis. (2001) 33:780–5. 10.1086/322669 11512082

[110] Steere AC. Lyme disease. N Engl J Med. (1989) 345:586–96. 10.1056/NEJM198908313210906 2668764

[111] Schutzer SE Body BA Boyle J Branson BM Dattwyler RJ Fikrig E. Direct diagnostic tests for Lyme disease. Clin Infect Dis. (2019) 68:1052–7. 10.1093/cid/ciy614 30307486 PMC6399434

[112] Hodzic E Feng S Holden K Freet KJ Barthold SW. Persistence of *Borrelia burgdorferi* following antibiotic treatment in mice. Antimicrob Agents Chemother. (2008) 52:1728–36. 10.1128/AAC.01050-07 18316520 PMC2346637

[113] Krause PJ. Human babesiosis. Int J Parasitol. (2019) 49:165–74. 10.1016/j.ijpara.2018.11.007 30690090

[114] Lantos PM Wormser GP. Chronic coinfections in patients diagnosed with chronic lyme disease: a systematic review. Am J Med. (2014) 127:1105–10. 10.1016/j.amjmed.2014.05.036 24929022 PMC4252587

[115] Angelakis E Billeter SA Breitschwerdt EB Chomel BB Raoult D. Potential for tick-borne bartonelloses. Emerg Infect Dis. (2010) 16:385–91. 10.3201/eid1603.081685 20202411 PMC3322042

[116] Horowitz RI Freeman PR. Precision medicine: retrospective chart review and data analysis of 200 patients on dapsone combination therapy for chronic Lyme disease/post-treatment Lyme disease syndrome: part 1. Int J Gen Med. (2019) 12:101–19. 10.2147/IJGM.S193608 30863136 PMC6388746

[117] Halperin JJ Krupp LB Golightly MG Volkman DJ. Lyme borreliosis-associated encephalopathy. Neurology. (1990) 40:1340–3. 10.1212/WNL.40.9.1340 2392213

[118] Fernandez RE Rothberg M Ferencz G Wujack D. Lyme disease of the CNS: MR imaging findings in 14 cases. AJNR Am J Neuroradiol. (1990) 11:479–81. 2112310 PMC8367489

[119] Halperin JJ Pass HL Anand AK Luft BJ Volkman DJ Dattwyler RJ. Nervous system abnormalities in Lyme disease. Ann N Y Acad Sci. (1988) 539:24–34. 10.1111/j.1749-6632.1988.tb31835.x 3190096

[120] Hildenbrand P Craven DE Jones R Nemeskal P. Lyme neuroborreliosis: manifestations of a rapidly emerging zoonosis. AJNR Am J Neuroradiol. (2009) 30:1079–87. 10.3174/ajnr.A1579 19346313 PMC7051319

[121] Aalto A Sjöwall J Davidsson L Forsberg P Smedby O Brain magnetic resonance imaging does not contribute to the diagnosis of chronic neuroborreliosis. Acta Radiol. (2007) 48:755–62. 10.1080/02841850701367903 17729007

[122] Morgen K Martin R Stone RD Grafman J Kadom N McFarland HF Marques A. FLAIR and magnetization transfer imaging of patients with post-treatment Lyme disease syndrome. Neurology. (2001) 57:1980–5. 10.1212/WNL.57.11.1980 11739813

[123] Donta ST Noto RB Vento JA. SPECT brain imaging in chronic Lyme disease. Clin Nucl Med. (2012) 37:e219–22. 10.1097/RLU.0b013e318262ad9b 22889796

[124] Fallon BA Keilp J Prohovnik I Heertum RV Mann JJ. Regional cerebral blood flow and cognitive deficits in chronic lyme disease. J Neuropsychiatry Clin Neurosci. (2003) 15:326–32. 10.1176/jnp.15.3.326 12928508

[125] Fallon BA Lipkin RB Corbera KM Yu S Nobler MS Keilp JG. Regional cerebral blood flow and metabolic rate in persistent Lyme encephalopathy. Arch Gen Psychiatry. (2009) 66:554–63. 10.1001/archgenpsychiatry.2009.29 19414715

[126] Lindland ES Solheim AM Andreassen S Quist-Paulsen E Eikeland R Ljøstad U. Imaging in Lyme neuroborreliosis. Insights Imaging. (2018) 9:833–44. 10.1007/s13244-018-0646-x 30187265 PMC6206375

[127] Coughlin JM Yang T Rebman AW Bechtold KT Du Y Mathews WB. Imaging glial activation in patients with post-treatment Lyme disease symptoms: a pilot study using [ ^11^ C]DPA-713 PET. J Neuroinflammation. (2018) 15:346. 10.1186/s12974-018-1381-4 30567544 PMC6299943

[128] Touradji P Aucott JN Yang T Rebman AW Bechtold KT. Cognitive decline in post-treatment Lyme disease syndrome. Arch Clin Neuropsychol. (2019) 34:455–65. 10.1093/arclin/acy051 29945190

[129] Westervelt HJ McCaffrey RJ. Neuropsychological functioning in chronic Lyme disease. Neuropsychol Rev. (2002) 12:153–77. 10.1023/A:1020381913563 12428915

[130] Keilp JG Corbera K Slavov I Taylor MJ Sackeim HA Fallon BA. WAIS-III and WMS-III performance in chronic Lyme disease. J Int Neuropsychol Soc. (2006) 12:119–29. 10.1017/S1355617706060231 16433951

[131] Pollina DA Sliwinski M Squires NK Krupp LB. Cognitive processing speed in Lyme disease. Neuropsychiatry Neuropsychol Behav Neurol. (1999) 12:72–8. 10082336

[132] Keilp JG Corbera K Gorlyn M Oquendo MA Mann JJ Fallon BA. Neurocognition in post-treatment Lyme disease and major depressive disorder. Arch Clin Neuropsychol. (2019) 34:466–80. 10.1093/arclin/acy083 30418507

[133] Kaplan RF Jones-Woodward L Workman K Steere AC Logigian EL Meadows ME. Neuropsychological deficits in Lyme disease patients with and without other evidence of central nervous system pathology. Appl Neuropsychol. (1999) 6:3–11. 10.1207/s15324826an0601_1 10382565

[134] Elkins LE Pollina DA Scheffer SR Krupp LB. Psychological states and neuropsychological performances in chronic Lyme disease. Appl Neuropsychol. (1999) 6:19–26. 10.1207/s15324826an0601_3 10382567

[135] Kaplan RF Trevino RP Johnson GM Levy L Dornbush R Hu LT. Cognitive function in post-treatment Lyme disease Do additional antibiotics help? Neurology. (2003) 60:1916–22. 10.1212/01.WNL.0000068030.26992.25 12821733

[136] Soloski MJ Crowder LA Lahey LJ Wagner CA Robinson WH Aucott JN. Serum inflammatory mediators as markers of human Lyme disease activity. PLoS ONE. (2014) 9:e93243. 10.1371/journal.pone.0093243 24740099 PMC3989169

[137] Feng J Li T Yee R Yuan Y Bai C Cai M. Stationary phase persister/biofilm microcolony of *Borrelia burgdorferi* causes more severe disease in a mouse model of Lyme arthritis: implications for understanding persistence, Post-treatment Lyme Disease Syndrome (PTLDS), and treatment failure. Discov Med. (2019) 27:125–38. 30946803

[138] Sharma B Brown AV Matluck NE Hu LT Lewis K. *Borrelia burgdorferi*, the causative agent of Lyme disease, forms drug-tolerant persister cells. Antimicrob Agents Chemother. (2015) 59:4616–24. 10.1128/AAC.00864-15 26014929 PMC4505243

[139] Crossland NA Alvarez X Embers ME. Late disseminated Lyme disease: associated pathology and spirochete persistence posttreatment in Rhesus Macaques. Am J Pathol. (2018) 188:672–82. 10.1016/j.ajpath.2017.11.005 29242055 PMC5840488

[140] Hodzic E Imai D Feng S Barthold SW. Resurgence of persisting non-cultivable *Borrelia burgdorferi* following antibiotic treatment in mice. PLoS ONE. (2014) 9:e86907. 10.1371/journal.pone.0086907 24466286 PMC3900665

[141] Straubinger RK Summers BA Chang YF Appel MJG. Persistence of *Borrelia burgdorferi* in experimentally infected dogs after antibiotic treatment. J Clin Microbiol. (1997) 35:111–6. 10.1128/JCM.35.1.111-116.1997 8968890 PMC229521

[142] Bockenstedt LK Mao J Hodzic E Barthold SW Fish D. Detection of attenuated, noninfectious spirochetes in *Borrelia burgdorferi*-infected mice after antibiotic treatment. J Infect Dis. (2002) 186:1430–7. 10.1086/345284 12404158

[143] Bockenstedt LK Gonzalez DG Haberman AM Belperron AA. Spirochete antigens persist near cartilage after murine Lyme borreliosis therapy. J Clin Invest. (2012) 122:2652–60. 10.1172/JCI58813 22728937 PMC3386809

[144] Whiteside SK Snook JP Ma Y Sonderegger FL Fisher C Petersen C. IL-10 deficiency reveals a role for TLR2-dependent bystander activation of T cells in Lyme arthritis. J Immunol. (2018) 200:1457–70. 10.4049/jimmunol.1701248 29330323 PMC5809275

[145] Carlson D Hernandez J Bloom BJ Coburn J Aversa JM Steere AC. Lack of *Borrelia burgdorferi* DNA in synovial samples from patients with antibiotic treatment-resistant Lyme arthritis. Arthritis Rheum. (1999) 42:2705–9. 10616021 10.1002/1529-0131(199912)42:12<2705::AID-ANR29>3.0.CO;2-H

[146] Steere AC Gross D Meyer AL Huber BT. Autoimmune mechanisms in antibiotic treatment-resistant Lyme arthritis. J Autoimmun. (2001) 16:263–8. 10.1006/jaut.2000.0495 11334491

[147] Jutras BL Lochhead RB Kloos ZA Biboy J Strle K Booth CJ. *Borrelia burgdorferi* peptidoglycan is a persistent antigen in patients with Lyme arthritis. Proc Natl Acad Sci USA. (2019) 116:13498–507. 10.1073/pnas.1904170116 31209025 PMC6613144

[148] Wormser GP Nadelman RB Schwartz I. The amber theory of Lyme arthritis: initial description and clinical implications. Clin Rheumatol. (2012) 31:989–94. 10.1007/s10067-012-1964-x 22411576

[149] Chandra A Wormser GP Klempner MS Trevino RP Crow MK Latov N. Anti-neural antibody reactivity in patients with a history of Lyme borreliosis and persistent symptoms. Brain Behav Immun. (2010) 24:1018–24. 10.1016/j.bbi.2010.03.002 20227484 PMC2897967

[150] Schutzer SE Angel TE Liu T Schepmoes AA Clauss TR Adkins JN. Distinct cerebrospinal fluid proteomes differentiate post-treatment Lyme disease from chronic fatigue syndrome. PLoS ONE. (2011) 6:e17287. 10.1371/journal.pone.0017287 21383843 PMC3044169

[151] Hickie I Davenport T Wakefield D Vollmer-Conna U Cameron B Vernon SD. Post-infective and chronic fatigue syndromes precipitated by viral and non-viral pathogens: prospective cohort study. BMJ. (2006) 333:575. 10.1136/bmj.38933.585764.AE 16950834 PMC1569956

[152] Batheja S Nields JA Landa A Fallon BA. Post-treatment Lyme syndrome and central sensitization. J Neuropsychiatry Clin Neurosci. (2013) 25:176–86. 10.1176/appi.neuropsych.12090223 24026711

[153] Rebman AW Aucott JN Weinstein ER Bechtold KT Smith KC Leonard L. Living in limbo: contested narratives of patients with chronic symptoms following Lyme disease. Qual Health Res. (2017) 27:534–46. 10.1177/1049732315619380 26631681

[154] Fallon BA Keilp JG Corbera KM Petkova E Britton CB Dwyer E. A randomized, placebo-controlled trial of repeated IV antibiotic therapy for Lyme encephalopathy. Neurology. (2008) 70:992–1003. 10.1212/01.WNL.0000284604.61160.2d 17928580

[155] Cameron D. Severity of Lyme disease with persistent symptoms. Insights from a double-blind placebo-controlled clinical trial. Minerva Med. (2008) 99:489–96. 18971914

[156] Evers AWM van den Hoogen FH Donders ART van Middendorp H Berende A ter Hofstede HJM. Randomized trial of longer-term therapy for symptoms attributed to Lyme disease. N Engl J Med. (2016) 374:1209–20. 10.1056/NEJMoa1505425 27028911

[157] Delong AK Blossom B Maloney EL Phillips SE. Antibiotic retreatment of Lyme disease in patients with persistent symptoms: a biostatistical review of randomized, placebo-controlled, clinical trials. Contemp Clin Trials. (2012) 33:1132–42. 10.1016/j.cct.2012.08.009 22922244

[158] Fallon BA Petkova E Keilp JG Britton CB. A reappraisal of the U.S. clinical trials of post-treatment Lyme disease syndrome. Open Neurol J. (2012) 6:79–87. 10.2174/1874205X01206010079 23091568 PMC3474942

[159] Marzec NS Nelson C Waldron PR Blackburn BG Hosain S Greenhow T. Serious bacterial infections acquired during treatment of patients given a diagnosis of chronic Lyme Disease — United States. Morb Mortal Wkly Rep. (2017) 66:607–9. 10.15585/mmwr.mm6623a3 28617768 PMC5657841

[160] Patel R Grogg KL Edwards WD Wright AJ Schwenk NM. Death from inappropriate therapy for Lyme disease. Clin Infect Dis. (2000) 31:1107–9. 10.1086/318138 11049799

[161] Auwaerter PG Point: antibiotic therapy is not the answer for patients with persisting symptoms attributable to Lyme disease. Clin Infect Dis. (2007) 45:143–8. 10.1086/518854 17578771

[162] Cameron DJ Johnson LB Maloney EL. Evidence assessments and guideline recommendations in Lyme disease: the clinical management of known tick bites, erythema migrans rashes and persistent disease. Expert Rev Anti Infect Ther. (2014) 12:1103–35. 10.1586/14787210.2014.940900 25077519 PMC4196523

[163] Shor S Green C Szantyr B Phillips S Liegner K Burrascano JJ. Chronic Lyme disease: an evidence-based definition by the ILADS working group. Antibiot. (2019) 8:269. 10.3390/antibiotics8040269 31888310 PMC6963229

[164] Zifko UA Rupp M Schwarz S Zipko HT Maida EM. Modafinil in treatment of fatigue in multiple sclerosis: results of an open-label study. J Neurol. (2002) 249:983–7. 10.1007/s00415-002-0765-6 12195441

[165] Haugmark T Hagen KB Smedslund G Zangi HA. Mindfulness- and acceptance-based interventions for patients with fibromyalgia - A systematic review and meta-analyses. PLoS ONE. (2019) 14:e0221897. 10.1371/journal.pone.0221897 31479478 PMC6719827

[166] D'Adamo CR McMillin CR Chen KW Lucas EK Berman BM. Supervised resistance exercise for patients with persistent symptoms of Lyme disease. Med Sci Sports Exerc. (2015) 47:2291–8. 10.1249/MSS.0000000000000683 25899100

[167] Feng J Zhang S Shi W Zubcevik N Miklossy J Zhang Y. Selective essential oils from spice or culinary herbs have high activity against stationary phase and biofilm *Borrelia burgdorferi*. Front Med. (2017) 4:169. 10.3389/fmed.2017.00169 29075628 PMC5641543

[168] Wu X Sharma B Niles S O'Connor K Schilling R Matluck N. Identifying vancomycin as an effective antibiotic for killing *Borrelia burgdorferi*. Antimicrob Agents Chemother. (2018) 62:e01201–18. 10.1128/AAC.01201-18 30126963 PMC6201113

[169] Pothineni VR Wagh D Babar MM Inayathullah M Solow-Cordero D Kim KM. Identification of new drug candidates against *Borrelia burgdorferi* using high-throughput screening. Drug Des Devel Ther. (2016) 10:1307–22. 10.2147/DDDT.S101486 27103785 PMC4827596

[170] Feng J Wang T Shi W Zhang S Sullivan D Auwaerter PG. Identification of novel activity against *Borrelia burgdorferi* persisters using an FDA approved drug library. Emerg Microbes Infect. (2014) 3:e49. 10.1038/emi.2014.53 26038747 PMC4126181

[171] Feng J Auwaerter PG Zhang Y. Drug combinations against *Borrelia burgdorferi* persisters *in vitro*: eradication achieved by using daptomycin, cefoperazone and doxycycline. PLoS ONE. (2015) 10:e0117207. 10.1371/journal.pone.0117207 25806811 PMC4373819

[172] Aucott JN. Posttreatment Lyme disease syndrome. Infect Dis Clin North Am. (2015) 29:309–23. 10.1016/j.idc.2015.02.012 25999226

[173] Arvikar SL Steere AC. Diagnosis and treatment of Lyme arthritis. Infect Dis Clin North Am. (2015) 29:269–80. 10.1016/j.idc.2015.02.004 25999223 PMC4443866

[174] Feder HM Jr Johnson BJ O'Connell S Shapiro ED Steere AC Wormser GP. A critical appraisal of “chronic Lyme disease.” N Engl J Med. (2007) 357:1422–30. 10.1056/NEJMra072023 17914043

[175] Dumes AA. Lyme Disease and the epistemic tensions of “medically unexplained illnesses”. Med Anthropol. (2019) 1–16. 10.1080/01459740.2019.1670175 31860363

[176] Nowakowski J McKenna D Nadelman RB Cooper D Bittker S Holmgren D. Failure of treatment with cephalexin for Lyme disease. Arch Fam Med. (2000) 9:563–7. 10.1001/archfami.9.6.563 10862221

[177] Smith IS Rechlin D. Delayed diagnosis of neuroborreliosis presenting as bell palsy and meningitis. JAOA J Am. (2010) 110:441–4. 20805550

[178] Tumminello R Glaspey L Bhamidipati A Sheehan P Patel S. Early disseminated Lyme disease masquerading as mononucleosis: a case report. J Emerg Med. (2017) 53:e133–5. 10.1016/j.jemermed.2017.09.005 29102094

[179] Pavletic AJ Marques AR. Early disseminated Lyme disease causing false-positive serology for primary Epstein-Barr virus infection: report of 2 cases. Clin Infect Dis. (2017) 65:336–7. 10.1093/cid/cix298 28379435 PMC5848374

[180] Aucott JN Crowder LA Yedlin V Kortte KB. Bull's-Eye and nontarget skin lesions of Lyme disease: an internet survey of identification of erythema migrans. Dermatol Res Pr. (2012) 2012:451727. 10.1155/2012/451727 23133445 PMC3485866

[181] Tibbles CD Edlow JA. Does this patient have erythema migrans? JAMA. (2007) 297:2617–27. 10.1001/jama.297.23.2617 17579230

[182] Mazori DR Orme CM Mir A Meehan SA Neimann AL. Vesicular erythema migrans: an atypical and easily misdiagnosed form of Lyme disease. Dermatol Online J. (2015) 21:5. 26437159

[183] Adrion ER Aucott J Lemke KW Weiner JP. Health care costs, utilization and patterns of care following Lyme disease. PLoS ONE. (2015) 10:e0116767. 10.1371/journal.pone.0116767 25650808 PMC4317177

[184] Johnson L Aylward A Stricker RB. Healthcare access and burden of care for patients with Lyme disease: a large United States survey. Health Policy. (2011) 102:64–71. 10.1016/j.healthpol.2011.05.007 21676482

[185] Johnson M Feder HM Jr. Chronic Lyme disease: a survey of Connecticut primary care physicians. J Pediatr. (2010) 157:1022–5. 10.1016/j.jpeds.2010.06.031 20813379

[186] Crowder LA Yedlin VA Weinstein ER Kortte KB Aucott JN. Lyme disease and post-treatment Lyme disease syndrome: the neglected disease in our own backyard. Public Health. (2014) 128:784–91. 10.1016/j.puhe.2014.06.016 25213101

[187] Lantos PM Shapiro ED Auwaerter PG Baker PJ Halperin JJ McSweegan E. Unorthodox alternative therapies marketed to treat Lyme disease. Clin Infect Dis. (2015) 60:1776–82. 10.1093/cid/civ186 25852124 PMC4490322

[188] Ali A Vitulano L Lee R Weiss TR Colson ER. Experiences of patients identifying with chronic Lyme disease in the healthcare system: a qualitative study. BMC Fam Pr. (2014) 15:79. 10.1186/1471-2296-15-79 24885888 PMC4012507

[189] Drew D Hewitt H. A qualitative approach to understanding patients' diagnosis of Lyme disease. Public Heal Nurs. (2006) 23:20–6. 10.1111/j.0737-1209.2006.230104.x 16460417

[190] Mechanic D Meyer S. Concepts of trust among patients with serious illness. Soc Sci Med. (2000) 51:657–68. 10.1016/S0277-9536(00)00014-9 10975226

[191] Lobo CP Pfalzgraf AR Giannetti V Kanyongo G. Impact of invalidation and trust in physicians on health outcomes in fibromyalgia patients. Prim Care Companion CNS Disord. (2014) 16. 10.4088/PCC.14m01664 25667809 PMC4321014

[192] Aronowitz RA. Lyme disease: the social construction of a new disease and its social consequences. Milbank Q. (1991) 69:79–112. 10.2307/3350122 2034186

